# Engineering 3D perovskites for photon interconversion applications

**DOI:** 10.1371/journal.pone.0230299

**Published:** 2020-03-19

**Authors:** Sarah Wieghold, Lea Nienhaus

**Affiliations:** Department of Chemistry and Biochemistry, Florida State University, Tallahassee, FL, United States of America; Georgia Institute of Technology, UNITED STATES

## Abstract

In this review, we highlight the current advancements in the field of triplet sensitization by three-dimensional (3D) perovskite nanocrystals and bulk films. First introduced in 2017, 3D perovskite sensitized upconversion (UC) is a young fast-evolving field due to the tunability of the underlying perovskite material. By tuning the perovskite bandgap, visible-to-ultraviolet, near-infrared-to-visible or green-to-blue UC has been realized, which depicts the broad applicability of this material. As this research field is still in its infancy, we hope to stimulate the field by highlighting the advantages of these perovskite nanocrystals and bulk films, as well as shedding light onto the current drawbacks. In particular, the keywords toxicity, reproducibility and stability must be addressed prior to commercialization of the technology. If successful, photon interconversion is a means to increase the achievable efficiency of photovoltaic cells beyond its current limits by increasing the window of useable wavelengths.

## 1. Introduction

Wavelength interconversion applications by photon upconversion (UC) bear great potential in a wide variety of optoelectronics ranging from photocatalysis, biological imaging, photovoltaics (PVs), low-cost night vision or infrared sensing and communications. Photon UC for PV applications has shown its greatest potential through the approach of triplet-triplet annihilation (TTA) due to the low incident light densities required, since the energy is stored in long-lived spin triplet states [1–4]. However, the largest drawback of these triplet states is that their direct optical excitation is ‘spin-forbidden’ and thus, it is difficult to directly excite them. To circumvent this issue, researchers have turned to a wide variety of triplet sensitizers ranging from metal-organic complexes with high spin-orbit coupling [5–13], semiconductor quantum dots [14–18] and more recently, lead halide perovskites [19–27] and other spin-mixing materials such as transition metal dichalcogenides [28,29]. While each of these approaches bears their own benefit, we will in the following focus on the emerging field of three-dimensional (3D) perovskite-sensitized UC.

Organic- and inorganic-based lead halide perovskites have exhibited unprecedented success in optoelectronics [30–33] such as PVs, light emitting diodes (LEDs), and more recently in photon interconversion applications [19,23,34–37]. Their successful application in optoelectronics is in large parts based on their rich photophysical properties such as long carrier lifetimes, high absorption cross sections, and compositional tunability of the bandgap. However, the unique advantages of lead halide perovskites, in particular, their high defect-tolerance [38,39] and facile solution-processability may also prove to be their biggest disadvantages in low-cost device fabrication where high repeatability and reproducibility are key to enabling up-scaling processes.

**Fig 1:**
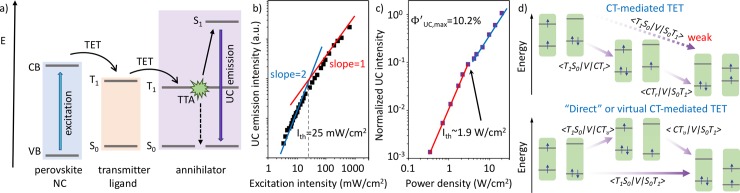
3D perovskite-sensitized UC. Visible-to-ultraviolet UC using CsPbX_3_ perovskite NCs to sensitize PPO via TTA. Reproduced with permission from Ref. [21]. Copyright 2019, The Chemical Society of Japan (CSJ). Green-to-blue UC using CsPbX_3_ perovskite NCs to sensitize DPA. Adapted from Ref. [19] with permission from The Royal Society of Chemistry. Near-infrared-to-visible UC using a bulk perovskite film to sensitize rubrene/DBP. Adapted from Ref. [23], Copyright 2019 Elsevier.

First introduced in 2017 [19], 3D perovskite-sensitized UC is a young fast-evolving field due to the tunability of the underlying perovskite material. For example, by tuning the perovskite bandgap, visible-to-ultraviolet [20,21], green-to-blue [19] or near-infrared-to-visible [22–24] UC has been realized which depicts the uniqueness of this absorber material. In this review, we will seek to highlight the recent advances in the field of 3D perovskite-based triplet sensitization (Fig 1). In particular, we will review the field in respect to nanocrystals (NCs) [19,20] and bulk film sensitization [22,23], discuss the main underlying processes for the creation of carriers and elucidate how they influence the energy transfer cascade resulting in UC.

In the last section of the review, we will speculate on further advancements in the field of 3D perovskite-sensitized UC. We noticed, that despite the recent advances in the field of 3D perovskite UC many research questions remain unanswered which currently hinder an implementation into existing technologies and prevent a scale-up of the technology. Drawbacks in the field can be summarized by the following key words: i) singlet fission, the reverse process of TTA, and singlet back-transfer to the sensitizer can limit the obtained UC efficiency in some annihilators, ii) trap states in the perovskite structure prevent an efficient energy transfer into the UC species, iii) optimizing the UC architecture and materials design to align energy levels of the perovskite and the annihilator for an efficient energy transfer, and iv) toxicity, long-term stability and reproducibility. We will critically discuss these issues which could in the future help to improve the current device UC architectures as well as could help material researchers to explore novel perovskite compositions and dimensionalities which can be used in UC applications.

### 1.1 Photon interconversion

Briefly, in photon UC higher energy photons are created from lower energy photons, effectively shortening the wavelength of the light emitted upon irradiation. To comply with energy conservation laws, photon UC relies on the combination of two or more low energy photons to one higher energy photon. While there are multiple routes to photon UC [40–42], we will focus on perovskite-sensitized triplet fusion (TF) UC in organic polyacenes, as this process bears the advantage of becoming efficient at sub-solar irradiances [19,23].

In sensitized TTA-UC two components are required: a sensitizer which is directly optically excited, and an annihilator, in which the triplet state is indirectly populated by a spin-allowed Dexter-type triplet energy transfer (TET) process or by direct charge injection. In the following, we will review the field and illuminate the use of 3D perovskite NCs and bulk films as sensitizers and discuss the different sensitization routes of the annihilator.

## 2. Review of the field

### 2.1 3D perovskite NCs-sensitized UC

**Fig 2:**
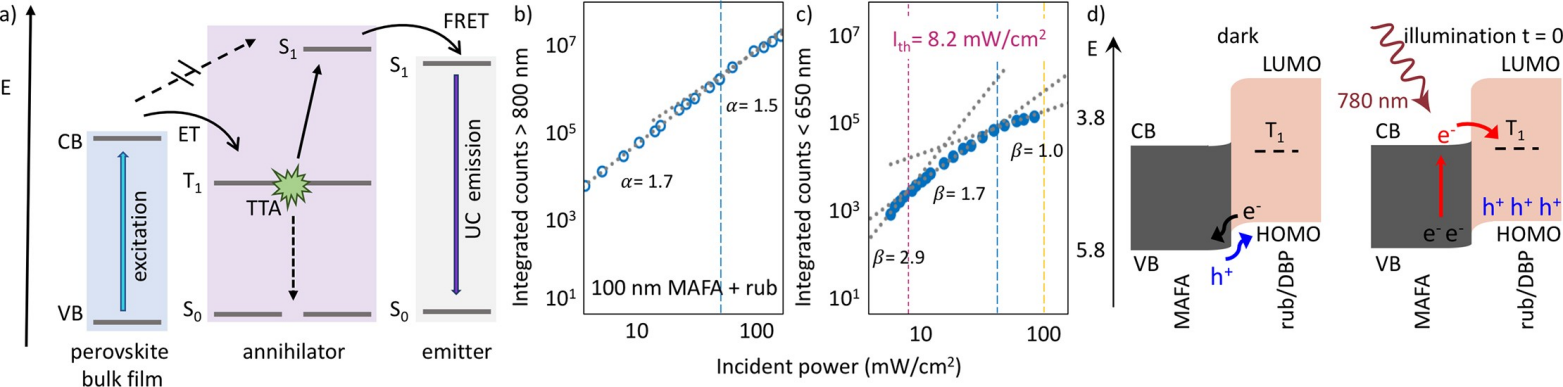
a) Schematic of TTA-UC using 3D perovskite NCs. b) Power-dependence of the upconverted emission of DPA/CsPbX_3_ (X = Br/I) NCs achieving green-to-blue UC with a low efficiency threshold of I_th_ = 25 mW/cm^2^. Adapted from Ref. [19] with permission from The Royal Society of Chemistry. c) Power-dependence of the UC emission using PPO/CsPbBr_3_ NCs exhibiting an efficiency threshold of I_th_ = 1.9 W/cm^2^ for visible-to-ultraviolet UC. An UC efficiency above 10% was reported. Adapted with permission from Ref. [20]. Copyright 2019 American Chemical Society. d) TET models via CT-mediated or ‘direct’/virtual CT-mediated TET in NCs. Adapted with permission from Macmillan Publishers Ltd.: Nature Communications from Ref. [43], Copyright 2020.

Fig 2A shows a schematic of the perovskite NCs-sensitized UC process via TTA. Solution-based 3D perovskite NCs-sensitized UC is based on TET from the perovskite NCs to a surface-bound transmitter ligand. A second TET step from the transmitter ligand to freely diffusing annihilator molecules populates the triplet state and enables the TTA-UC process. The first demonstration of this process using 3D perovskite NCs-sensitized UC was reported in 2017, where Kimizuka and co-workers [19] employed quantum-confined 3D CsPbX_3_ (X = Br/I) perovskite NCs to sensitize 9,10-diphenylanthracene (DPA), achieving green-to-blue UC with a low efficiency threshold *I*_*th*_ = 25 mW/cm^2^ (Fig 2B)_._ Wu and co-workers investigated the triplet sensitization mechanism of 1-pyrenecarboxylic acid (PCA) by varying the size of CsPbBr_3_ perovskite NCs finding a strong correlation between the NC size and the TET rate [26].

Concurrent studies of both groups resulted in the first demonstration of efficient visible-to-ultraviolet UC [20,21]. 3D perovskite NCs (CsPb(Cl/Br)_3_ and CsPbBr_3_) were used by both groups to sensitize triplet states in 2,5-diphenyloxazole (PPO) using a transmitter ligand based on 1-naphthalene carboxylic acid (NCA). Whereas Kimizuka and co-workers tuned the NC composition, Wu and co-workers studied the influence of NCs size on the UC properties. A similar efficiency threshold for the UC emission was calculated by both groups of 4.7 W/cm^2^ and 1.9 W/cm^2^ for the CsPb(Cl/Br)_3_ and CsPbBr_3_ NC systems, respectively (compare Fig 2C). A record visible-to-ultraviolet UC efficiency above 10% was reported for these 3D perovskite NCs systems [20].

In 2020, Castellano/Wu and co-workers [43] investigated the transfer mechanism across the inorganic perovskite NC/organic molecule interface. They used NCA and 5-tetracene carboxylic acid (TCA) as triplet acceptors to investigate the TET mechanism. As previously reported, naphthalene triplets can be populated via direct bound TET from CsPbBr_3_ NCs.[25] On the other hand, the band alignment in the case of TCA would allow for either a direct TET mechanism, or a hole transfer to a charge transfer state (CT) and subsequent electron transfer to populate the triplet state. Due to the fact that hole transfer outcompetes both exciton recombination and hole trapping, the CT-mediated TET is found to be the preferred sensitization pathway in the case of exothermic hole transfer. The two TET mechanisms are summarized in Fig 2D.

### 2.2 3D perovskite bulk film-sensitized UC

3D bulk perovskite films were first introduced as light-harvesting antennas to funnel energy into PbS quantum dots (QDs), which acted as spin-mixers to sensitize the triplet state of rubrene, allowing for UC to occur [27]. This approach was used to increase the limited absorption possible in QD-based solid-state devices [17,18]. In this study, methylammonium (MA) tin triiodide (MASnI_3_) was used in a trilayer device structure: MASnI_3_/PbS/rubrene.

Encouraged by the sub-bandgap onset of electroluminescence of rubrene-based organic light-emitting diodes (OLEDs), lead halide perovskite thin films based on methylammonium formamidinium (FA) lead triiodide (MAFA) were then investigated by our group as direct rubrene triplet sensitizers due to their favorable band alignment for direct injection into the rubrene triplet state, foregoing the quantum dot layer as a spin mixer [22–24,44]. This novel bilayer architecture bears three-fold advantages over previous approaches: a fully solution-processed device is possible for the first time, no surface-bound transmitter ligands are required and triplet generation occurs through free carrier injection. This carrier injection pathway removes the requirement of excitonic spin-mixing, and rather allows for a very high electron transfer yield, as strong spin-orbit coupling in the perovskite film will scramble the electron spin, enabling the electron to adopt the proper spin required for triplet generation.

**Fig 3:**
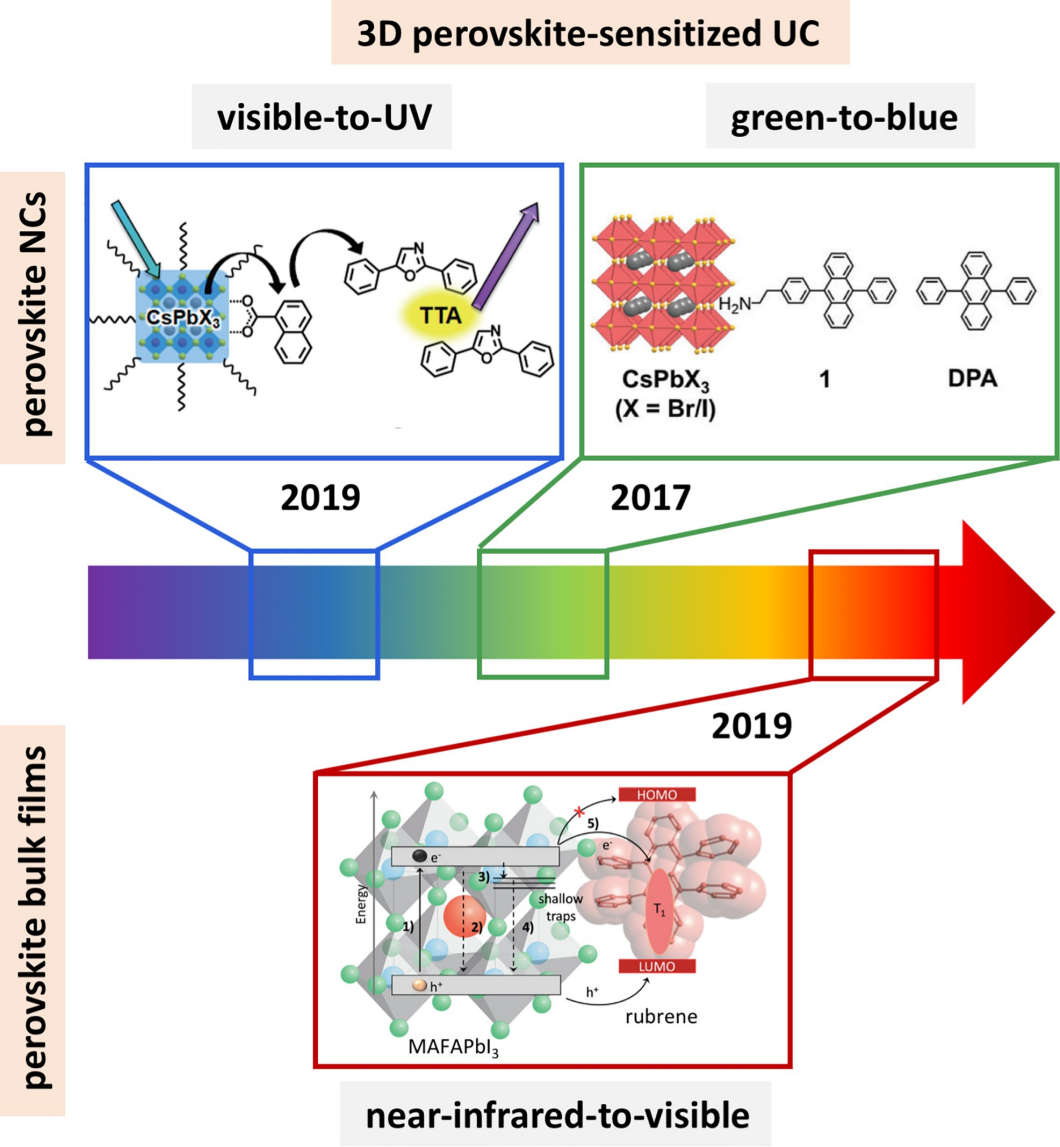
a) Schematic of photon UC using perovskite bulk films. b), c) Power dependence of the underlying perovskite PL (b) and UC emission (c) for a 100 nm thick perovskite film with a rubrene/DBP layer as upconverted on top. Adapted from Ref. [23], Copyright 2019 Elsevier. d) Band alignment diagram of a perovskite—rubrene interface. Adapted with permission from Ref. [44]. Copyright 2020, American Chemical Society.

Triplet-sensitization by free carrier injection is an emerging field, and as the field matures, more unanswered questions are solved, shedding light on the mechanism of TTA-UC in these devices [45]. Thus far, it has been established that the triplets stem from non-geminate charge carriers [23], and UC occurs via TTA (Fig 3A) [22].

Perovskite thickness-dependent studies show the underlying perovskite recombination dynamics directly dictate the UC process and trap filling and triplet sensitization are competing processes (Fig 3B). Sub-solar efficiency thresholds have been observed [23], potentially enabling real-world applications of solid-state UC (Fig 3C) in PVs. Two rates of triplet generation have been observed, which can be traced back to slow diffusion-mediated TTA far from the interface and rapid TTA close to the perovskite/rubrene interface, leading to a triplet-population dependent UC efficiency [24]. Lastly, an unusual effect occurs in these UC devices. Upon initial illumination, there is a rapid ‘photobleach’ of the upconverted light intensity [24], followed by a plateau of the UC efficiency, determined by the underlying perovskite recombination dynamics. Recent results have been able to attribute this effect to a space charge region created in the dark due to band bending at the interface (Fig 3D). This space charge region enables a high triplet generation upon initial illumination which diminishes until a new steady-state has been achieved [44]. Careful band engineering may be able to yield devices which can readily take advantage of this space charge region, boosting the overall performance.

The main advantage of employing bulk perovskite sensitizers over quantum-confined NC approaches is that the sensitization mechanism relies on free carrier injection. Charge transfer and diffusion are known to be faster than excitonic processes [46–48], allowing the triplet sensitization to outcompete rapid singlet backtransfer [17,49]. This enables the implementation of more optically dense sensitizers than in previously reported PbS NC-based solid-state devices, where the near-infrared (NIR) absorption was limited to <1%.

## 3. Outlook

### 3.1 Current shortcomings

**Fig 4:**
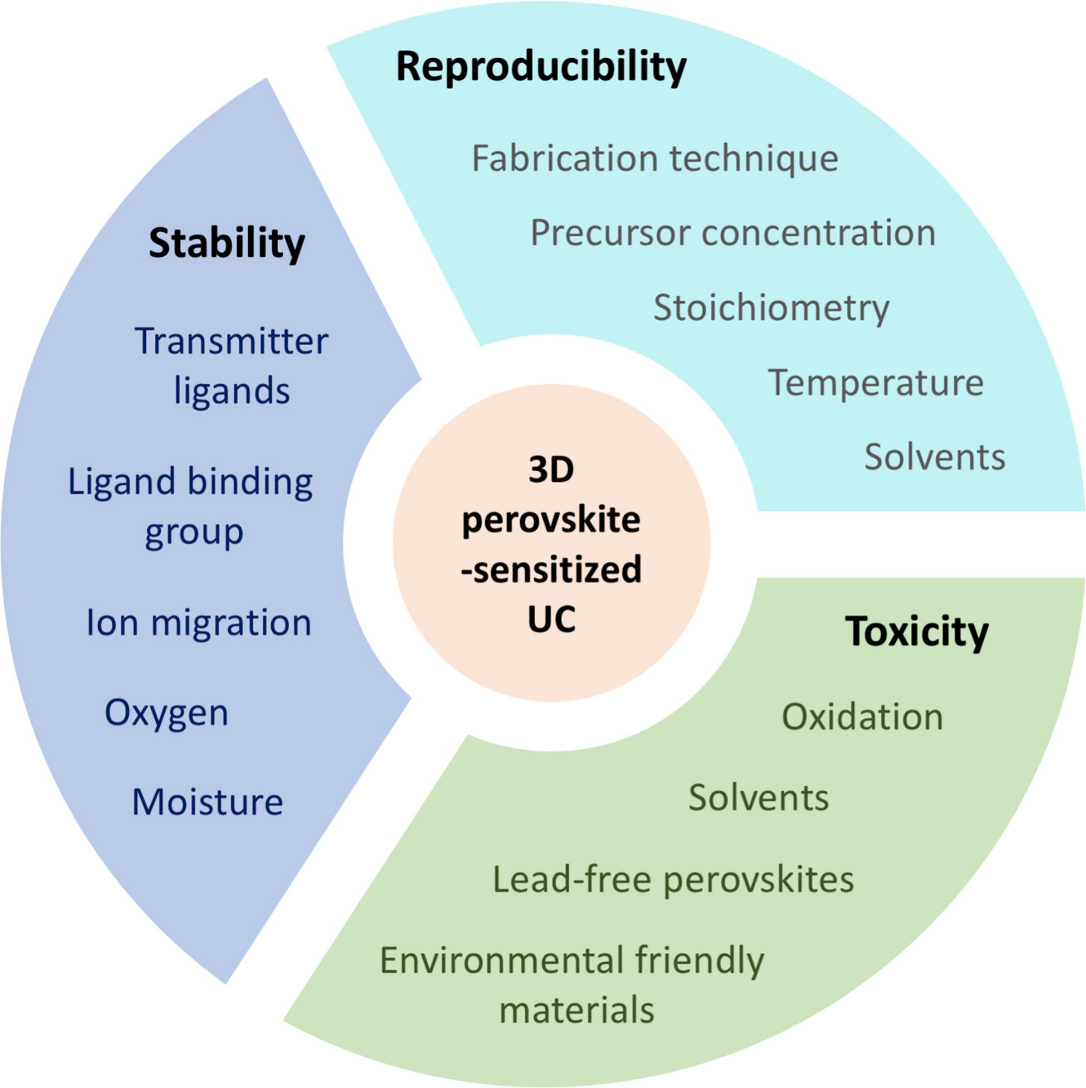
Overview of the current shortcomings of 3D perovskite-sensitized UC.

As common in all immature technologies, there are still a vast number of issues in 3D perovskite-sensitized UC that must be addressed prior to commercialization. Most importantly, issues of reproducibility, device inhomogeneity, long-term device stability and toxicity are crucial to the future of perovskite-sensitized TTA-UC (Fig 4).

#### 3.1.1 Reproducibility, inhomogeneities and stability

The properties of solution-fabricated perovskite NCs are highly dependent on the fabrication conditions. Slight variations in the precursor concentration or injection temperature can influence the particle size and shape thus, result in changes in the optoelectronic properties of 3D perovskite NCs. The ligand binding strength and ligand binding groups chosen, as well as the solvent can influence the underlying perovskite surface trap density. Work by Wu and co-workers has shown that quantum confinement in lead halide perovskite NCs enables the triplet sensitization process [26]. As a result, slight variations in particle size and energetics of the NCs can greatly influence the energy transfer to the triplet state. Furthermore, due to the bulky ligands required to stabilize the particles and passivate surface trap states in such NCs, tightly bound transmitter ligands are required to facilitate an efficient triplet transfer to the surrounding triplet acceptors.

Contrary, in bulk perovskite sensitization schemes, there is no need for stabilizing ligands. The film quality depends on the precursor concentration and stoichiometry, fabrication technique as well as the annealing time and temperature. While this inherent issue is able to be mitigated by careful fabrication practices, slight changes in the ambient environment, e.g. pressure, moisture or temperature are much more difficult to control precisely. As a result, perovskite films with slightly different trap densities are created with each device fabrication.

As of now, successful TTA-UC schemes have been reported in solution and as solid-state devices for perovskite NCs and bulk perovskite films, respectively. In solution-based approaches using perovskite NCs, one major drawback is related to the required ligands. These ligands are only weakly adsorbed to the surface, and an excess is needed in solution to maintain equilibrium conditions. Upon purification, ligands are commonly removed exposing surface defects which in turn reduce the PL quantum yield (QY) [50]. Recently, a huge research effort was centered to enhance the stability of these perovskite NCs. Approaches are based on surface manipulation which would allow the use of perovskite NCs without the need for passivating shells [51–54] or zwitterionic and bifunctional ligands [55,56]. Further, ‘bulk’ films made of NCs would be desirable for the use in solid-state applications. However, using approaches of shorter ligand moieties to form stable inks [54] in addition to bridging the NCs electronically [57,58] would be prerequisite.

For solid-state device fabrication, usually the bulk perovskite film as well as the upconverting species are solution-processed. However, this additional second solution-processed step to deposit the organic upconverter introduces additional trap states in the perovskite film, which is likely due to a slight dissolution of the topmost perovskite layer [44]. Although the addition of organic molecules is able to passivate surface trap states by non-covalent interactions, the exact interactions between the organic and the perovskite cannot be controlled. To address this issue, it will be beneficial to investigate the difference in the UC properties of the current generation of solution-processed devices, post-fabrication annealed devices, as well as devices in which the organic layer is thermally evaporated as in previous QD-based devices [17,59]. This may shed additional light on the role of surface trap states, as well as give insight on the importance of the packing of the organic layer, as this could be tuned by thermal annealing.

Additionally, one of the main unknowns in such solid-state UC devices is related to perovskite film inhomogeneities which are introduced during the fabrication process [60,61] or by the migration of ions. Upon illumination, the ions in this soft material can migrate, changing the local properties. In particular, this effect is known to change the bandgap of mixed-halide perovskites, which can greatly influence the local band structure and bandgap. In addition, time- and intensity-dependent trap filling and resulting ‘self-healing’ effects make it difficult to draw direct conclusions with regards to the UC efficiency. We have shown in the past that the UC efficiency is dependent on both the power of illumination and the time after illumination the measurement is taken [24]. As a result, a detailed measurement protocol, similar to the standardized photovoltaic power conversion efficiency (PCE) measurement is required. This effect may also indicate that the UC devices would benefit from long-term illumination, as would occur under daily use in a photovoltaic cell.

#### 3.1.2 Long-term stability

(Long-term) stability is an inherent issue of lead halide perovskites for both NCs and bulk films. Here, instability issues can be divided into the following sub-groups: hygroscopicity, thermal instability, and ion migration [62,63]. Under ambient conditions, perovskites decompose into PbI_2_ and the organic counterparts, due to their sensitivity to oxygen and moisture. To address this, perovskites are commonly encapsulated under air-free conditions, e.g. in a glovebox. However, even encapsulated, solid-state UC devices slowly degrade over time, which in turn can be correlated to a loss in efficiency. Approaches to stabilize the perovskite film structure are related to a substitution or doping of A-, B-, or X-site ions [64–68], or engineering of defects, interface and microstructure [69–71].

Similarly, solution-based perovskite NCs are inherently unstable, in particular, redder iodide-exchanged perovskite NCs readily degrade into non-emissive species. To address this NC degradation, an inorganic metal oxide shell [72] can be included, or improved ligands as shown by Kovalenko and co-workers [56,73]. Further, engineering of crystal symmetry and dimensionality could improve the long-term stability by forming ‘quasi-2D’ perovskites [74–76].

Air-free conditions are also crucial to the UC process, as the desired triplet states are rapidly quenched by oxygen (^3^O_2_), due to the diradical nature of the ground state of the oxygen molecule.

$$T_{1}+{}^{3}O_{2}\;\to \;S_{0}+{}^{1}O_{2}{}^{*}$$

The triplet state T_1_ is quenched to form the singlet ground state S_0_, while the triplet oxygen ^3^O_2_ is excited to its singlet state ^1^O_2_* [77,78]. Long-term stability measurements have been performed for perovskites, yet are still lacking in the field of perovskite-sensitized UC. In contrast to previously fabricated PbS QD-based devices, which degraded rapidly [17,79], the current generation of perovskite-sensitized are stable for up to several months. Future efforts will be directed at understanding the changes in the device properties over time, both with respect to the perovskite and organic semiconductor degradation.

#### 3.1.3 Toxicity

One of the largest issues facing the implementation of lead halide perovskites into industrial relevant devices is the water-solubility and toxicity of the materials used. While there is a large push in perovskite-based photovoltaics to become lead-free, currently there have only been reports of lead-based perovskites in UC applications. This is in great part due to the inherent instability of e.g. tin-based halide perovskites [80–82], but more importantly due to the absolute band energies of the materials. Lead-based perovskites have the ‘correct’ band alignment for exothermic hole transfer, as well as slightly exothermic electron transfer. The desired low bandgap tin-based perovskites have shallower valence and conduction bands [83], which disallow free carrier sensitization, and would only enable triplet sensitization by bound excitons. However, excitons rapidly dissociate in these materials, therefore this pathway is not expected to be efficient. Further investigation into Ruddlesden-Popper type perovskite-inspired materials [84–86] and less toxic double perovskites, combined with machine learning approaches [87–90] to find suitable materials has the potential to break the field wide open. In sensitized UC, not only the perovskite can be tuned, rather, we can also tune the energetics of the upconverting species. Electron withdrawing or electron donating side-groups on the polyacene backbone can vary the highest occupied molecular orbital (HOMO) and lowest unoccupied molecular orbital (LUMO) alignment, thus, allowing us to create a vast library of upconverting molecules which can be tailored to the specific perovskite of interest.

### 3.2 Future advancements

As mentioned previously, 3D perovskite-sensitized UC is an immature field. We hope to stimulate the field by highlighting both the advancements and current drawbacks. One of the greatest future advancements in perovskite-sensitized UC would be to increase the gains achievable by UC and shift the wavelengths to higher or lower energies. Currently, we are able to upconvert ~800 nm light to ~600 nm using lead iodide bulk perovskites films. However, in photovoltaic or imaging applications, it is desirable to upconvert light beyond the silicon bandgap of 1.1 eV. We have been able to show efficiency thresholds of less than 8 mW/cm^2^ at 780 nm, enabling the inclusion of UC into wider bandgap photovoltaics such as bromide-based perovskites under solar fluxes. The broad-band absorption of the perovskites will enable the capture of the 2.3–1.55 eV region of the solar spectrum. However, this broad-band absorption also poses the greatest drawback of these UC devices: after successful TTA in the organic layer, emission and reabsorption compete. Therefore, the perovskite film thickness must be closely tailored to the desired application.

As with all immature technologies, there is room to grow, and the current benchmark of the UC efficiency of ~3% for bulk perovskite sensitization is likely to be overcome soon by innovative engineering approaches.

Perovskite NCs bear their greatest potential in high-energy UC approaches, e.g. for catalytic applications or stable blue LEDs. By using tightly bound transmitter ligands, and state-of-the-art zwitterionic ligands to reduce the surface trap states further advancements are near. Dimerized annihilators can further boost the UC efficiency by increasing the probability of TTA within the triplet lifetime [91,92]. As the emission wavelength of perovskite NCs can be tuned up to ~3 eV, UC deep into the UV is feasible. Most current NC-based UC has been reported from green-to-blue, this is likely due to readily available light sources, as well as facile synthesis of CsPbBr_3_ NCs. However, perovskite NCs are readily tunable into the NIR, by halide exchange. Reports of high QY MAPbI_3_ and FAPbI_3_ could easily enable red-to-blue UC with minimal energy losses due to energetic offsets of the bandgap and the triplet energy.

To reduce toxicity, it will be beneficial to create a vast library of perovskite band energy levels and cross correlate these with the HOMO and LUMO levels of possible annihilators by a combination of theoretical and machine learning approaches. However, drawbacks of toxicity can readily be addressed by careful encapsulation measures, which are required to maintain the air-free environment desirable for efficient UC.

## 4. Conclusions

3D perovskite-sensitized UC bears a promising new approach to wavelength interconversion. While an immature field at present, the possibilities in applications are vast. Over the course of the last year, there have been many advances in 3D perovskite-based UC. For the first time, an efficiency threshold of under 10 mW/cm^2^ has been reported, coupled with an achievable film thickness which can absorb up to 60% of the incident light,[23] this paves the path towards real world applications.

Due to the wide tunability of halide perovskites, wavelength interconversion applications from visible-to-ultraviolet to infrared-to-visible can be envisioned. Charge carrier-based triplet sensitization in such bulk perovskite-based devices foregoes the previous requirement of efficient spin mixers, which may be able to boost the overall UC efficiency. Enhancements in the stability of both solution-based NC sensitized UC, and bulk-sensitized UC pave a path towards industrial applications of the technology.
