# Correction: Engineering 3D perovskites for photon interconversion applications

**DOI:** 10.1371/journal.pone.0232196

**Published:** 2020-04-16

**Authors:** Sarah Wieghold, Lea Nienhaus

The images for Figs 1, 2 and 3 are incorrectly switched. The image that appears as Fig 1 should be Fig 2, the image that appears as Fig 2 should be Fig 3, and the image that appears as Fig 3 should be Fig 1. The figure captions appear in the correct order. The authors have provided corrected versions here.

**Fig 1:**
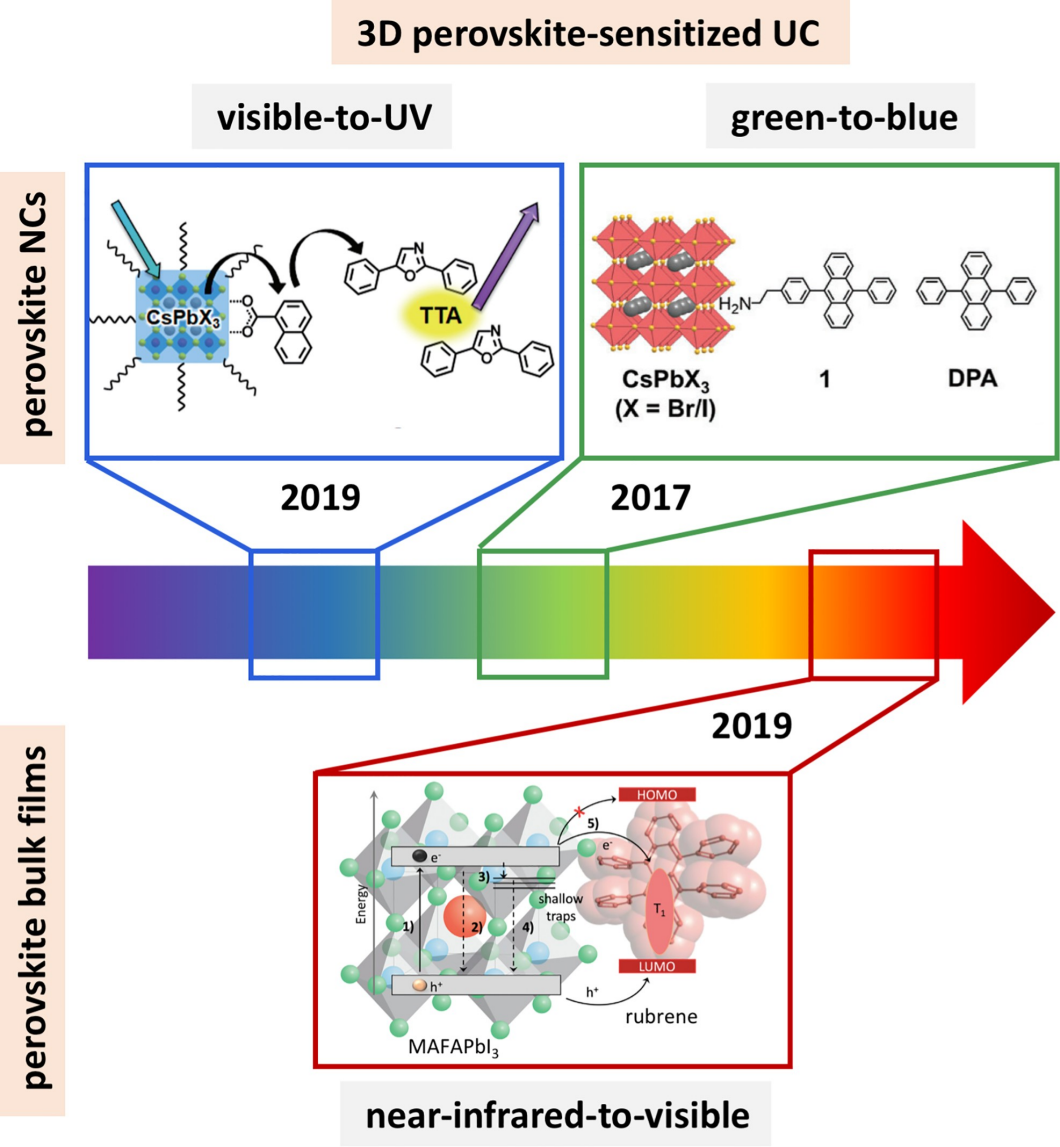
3D perovskite-sensitized UC. Visible-to-ultraviolet UC using CsPbX3 perovskite NCs to sensitize PPO via TTA. Reproduced with permission from Ref. [21]. Copyright 2019, The Chemical Society of Japan (CSJ). Green-to-blue UC using CsPbX3 perovskite NCs to sensitize DPA. Adapted from Ref. [19] with permission from The Royal Society of Chemistry. Near-infrared-to-visible UC using a bulk perovskite film to sensitize rubrene/DBP. Adapted from Ref. [23], Copyright 2019 Elsevier.

**Fig 2:**
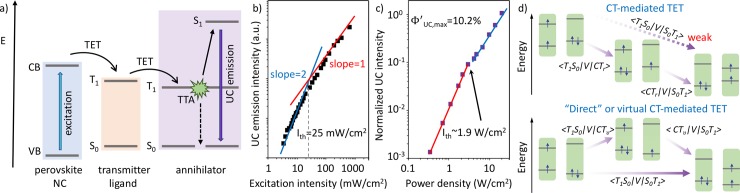
a) Schematic of TTA-UC using 3D perovskite NCs. b) Power-dependence of the upconverted emission of DPA/CsPbX3 (X = Br/I) NCs achieving green-to-blue UC with a low efficiency threshold of Ith = 25 mW/cm2. Adapted from Ref. [19] with permission from The Royal Society of Chemistry. c) Power-dependence of the UC emission using PPO/CsPbBr3 NCs exhibiting an efficiency threshold of Ith = 1.9 W/cm2 for visible-to-ultraviolet UC. An UC efficiency above 10% was reported. Adapted with permission from Ref. [20]. Copyright 2019 American Chemical Society. d) TET models via CT-mediated or ‘direct’/virtual CT-mediated TET in NCs. Adapted with permission from Macmillan Publishers Ltd.: Nature Communications from Ref. [43], Copyright 2020.

**Fig 3:**
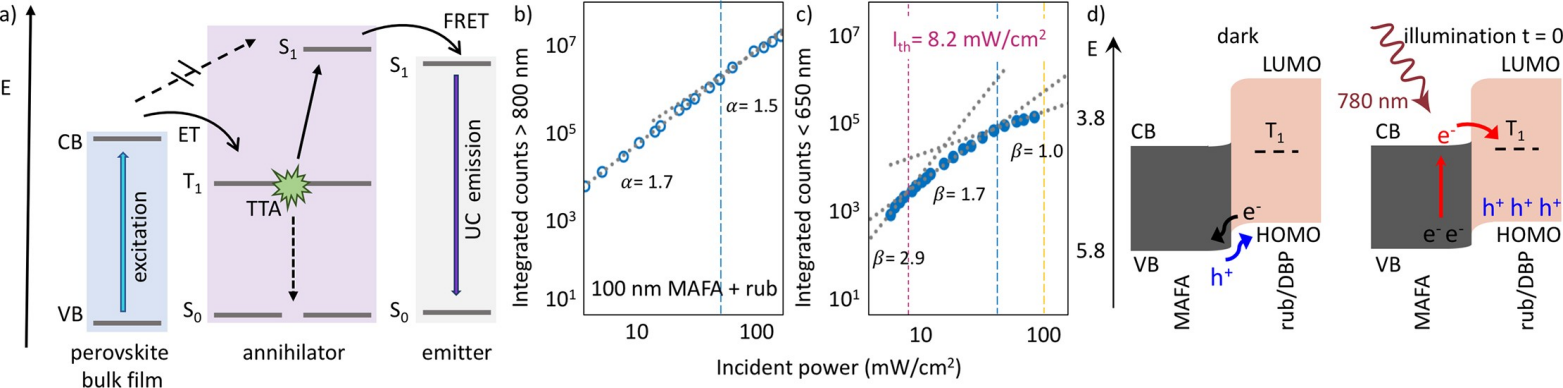
a) Schematic of photon UC using perovskite bulk films. b), c) Power dependence of the underlying perovskite PL (b) and UC emission (c) for a 100 nm thick perovskite film with a rubrene/DBP layer as upconverted on top. Adapted from Ref. [23], Copyright 2019 Elsevier. d) Band alignment diagram of a perovskite—rubrene interface. Adapted with permission from Ref. [44]. Copyright 2020, American Chemical Society.
